# Imaging of cerebrovascular complications from blunt skull base trauma

**DOI:** 10.1007/s10140-024-02243-z

**Published:** 2024-05-28

**Authors:** James Bai, Rahim Ismail, Alex Kessler, Daniel Kawakyu-O’Connor

**Affiliations:** 1 Department of Imaging Sciences, University of Rochester Medical Center, Rochester, NY USA; 2 School of Medicine and Dentistry, University of Rochester Medical Center, 601 Elmwood Ave, Box 648, 14642 Rochester, NY USA

**Keywords:** Blunt cerebrovascular injury, Skull base trauma, Anterior cranial fossa dural arterial venous fistula, Post-traumatic ICA dissection, Post-traumatic aneurysms, Carotidcavernous fistula, Blunt vertebral artery injury, Dural venous sinus thrombosis

## Abstract

Cerebrovascular complications from blunt trauma to the skull base, though rare, can lead to potentially devastating outcomes, emphasizing the importance of timely diagnosis and management. Due to the insidious clinical presentation, subtle nature of imaging findings, and complex anatomy of the skull base, diagnosing cerebrovascular injuries and their complications poses considerable challenges. This article offers a comprehensive review of skull base anatomy and pathophysiology pertinent to recognizing cerebrovascular injuries and their complications, up-to-date screening criteria and imaging techniques for assessing these injuries, and a case-based review of the spectrum of cerebrovascular complications arising from skull base trauma. This review will enhance understanding of cerebrovascular injuries and their complications from blunt skull base trauma to facilitate diagnosis and timely treatment.

## Introduction

Skull base fractures are defined as fractures involving the floor of the anterior, middle, or posterior cranial fossae, constituting a broad category of injuries owing to complex local anatomic relationships, irregular skull geometry, and differences in material properties of the skull base, its contents, and adjacent structures. Fractures can result from direct impact or inertial loading force, resulting in injury from local interaction with bones, ligaments, muscles, and neurovascular structures in the basilar skull [1].

Intracranial arterial vessels are more susceptible to rotational and shear forces because they are thinner and stiffer than extracranial segments, attributed to the absence of an external elastic lamina and the presence of a stiffer internal elastic lamina. Differences in material properties of the adjacent structures of the skull base and resulting differences in momentum can lead to arterial dissection, post-traumatic aneurysm formation, and the creation of dural arterial-venous fistulae. While vascular complications of the cervical vessels may also arise from skull base trauma due to shear and rotational forces, this review primarily focuses on intracranial cerebrovascular injuries.

Skull base fractures have been reported in 3.5 to 24% of overall skull fractures and 4% of overall head injuries [2]. Motor vehicle collisions are the most prevalent cause of skull base injuries, with the majority of injuries resulting from direct impact with rigid objects [3]. Cerebrovascular injuries have been detected in 8.5% of cases involving blunt skull base fractures, and there is a strong correlation with fractures that affect the sella turcica-sphenoid sinus complex, petrous carotid canal, and occipital condyles [4–6]. The risk for stroke attributable to blunt cerebrovascular injury ranges from 1 to 10% [7–9]. For patients with higher-grade vessel injuries, the risk can increase as much as 33%, with mortality rates ranging from 15 to 59% [9, 10].

Blunt cerebrovascular injury can be stratified using a five-point Biffle scale based on the severity of vessel injury, ranging from mild intimal injury (Grade I) to vessel transection (Grade V) (Table 1) [11]. Grade I injuries tend to spontaneously resolve in two-thirds of cases, whereas Grade V injuries are almost universally fatal [4, 11]. High-grade injuries are more likely to deteriorate compared to low-grade injuries and are associated with higher rates of stroke [9, 12].

**Table 1:**
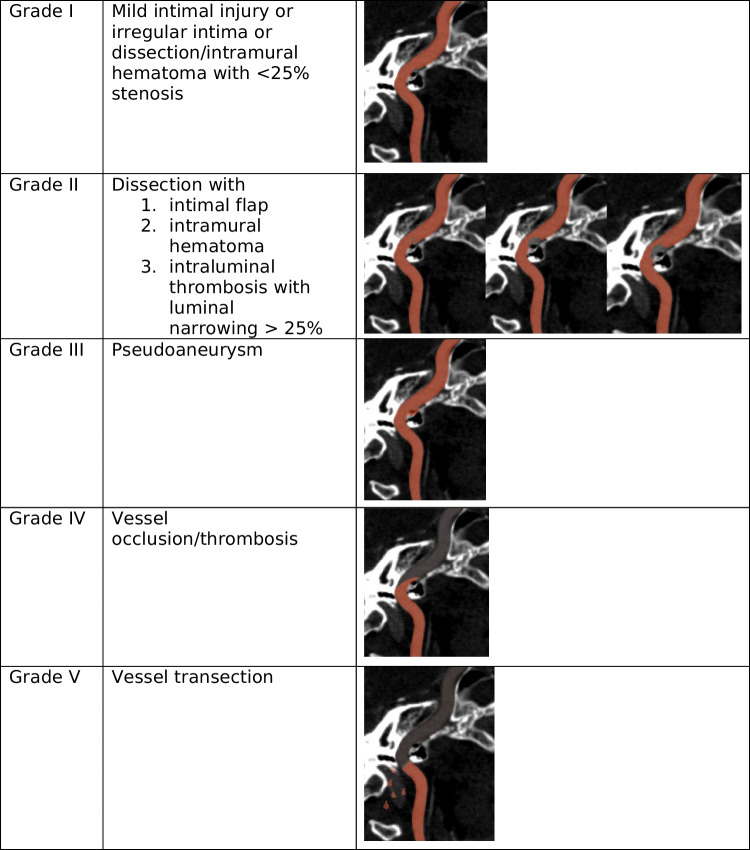
Biffl scale for blunt cerebrovascular injury

Imaging plays a crucial role in the evaluation of vascular injuries resulting from skull base trauma. Computed Tomography Angiogram (CTA), with its rapid acquisition, high diagnostic accuracy, and wide availability, is the primary non-invasive imaging modality in the setting of suspected cerebrovascular injury. Magnetic Resonance Imaging (MRI)/Magnetic Resonance Angiography (MRA) and vessel wall image (VWI) can offer additional diagnostic value in equivocal cases and provide better assessment of cerebral ischemic infarction associated with cerebrovascular injury. Digital Subtraction Angiography (DSA), with its superior spatial and temporal resolution, remains the gold standard for diagnosing cerebral vascular injuries. Duplex ultrasound is not the preferred choice for evaluating traumatic cerebrovascular injuries and is not recommended by the EAST guidelines [13], primarily due to its low sensitivity for detecting vessel dissection, difficulty in sonographically accessing the vascular segments at the skull base.

### Relevant anatomy

The skull base is classically divided into three regions: the anterior cranial fossa (ACF), which comprises the orbital plate of the frontal bone, the lesser wings of the sphenoid, and the cribriform plate of ethmoid bone; the middle cranial fossa (MCF), including the greater wing of the sphenoid and the temporal bone; and the posterior cranial fossa (PCF), primarily composed of the occipital bone and the squamous and mastoid portions of the temporal bone. The skull base has various foramina and canals that transmit important neurovascular structures. In this review, we will focus our discussion on the skull base structures that are most relevant to traumatic cerebrovascular injuries (Table 2).

**Table 2:** Skull base structures that are most susceptible to cerebrovascular injuries

	Important skull base canals, foramina, and grooves housing vascular structures	Vascular structures	Commonly associated cerebrovascular complications from trauma
Anterior Cranial Fossa	Anterior and posterior ethmoidal foramen	Anterior and posterior ethmoidal artery	Dural AV Fistula
	Anterior caudal aspect of groove of superior sagittal sinus	Superior sagittal sinus (Anterior attachment site)	Venous injury
Middle Cranial Fossa	Carotid Canal	ICA (Petrous segment)	Dissection, pseudoaneurysm
	Foramen lacerum	ICA (Lacerum segment) – course above the foramen without passing through.	Dissection, pseudoaneurysm
	Carotid sulcus, sella turcica-sphenoid sinus complex	ICA (Cavernous segment) – Sit on the carotid sulcus lateral to the sella turcica	Dissection, pseudoaneurysm
		Cavernous sinus	Carotid cavernous fistula
	Anterior clinoid process (lateral to the clinoid the ICA) and carotid sulcus (medial to clinoid ICA)	ICA (Clinoid, ophthalmic segments) – Course between the anterior clinoid process and the carotid sulcus	Dissection, pseudoaneurysm
	Optic canal (Running through the lesser wing of the sphenoid medial to the anterior clinoid process)	Ophthalmic artery	Dissection, pseudoaneurysm
Posterior Cranial Fossa	Foramen magnum	Vertebral arteries	Dissection, pseudoaneurysm
	Sigmoid groove	Sigmoid sinus	Dural venous sinus thrombosis

#### Anterior cranial fossa

The ACF is defined laterally by the orbital plate of the frontal bone, medially by the cribriform plate of the ethmoid bone, and posteriorly by the tuberculum sellae and the lesser wing of the sphenoid bone. The anterior ethmoidal artery, a branch of the ophthalmic artery, enters the anterior cranial fossa through the ethmoidal foramen, which is a small opening in the cribriform plate of the ethmoid bone. This region is susceptible to fracture of the cribriform plate, which can result in injuries to the anterior ethmoidal artery. The anterior caudal aspect of the groove of the superior sagittal sinus merges at midline with the anterior cranial fossa from the frontal crest. This region serves as the anterior attachment site for the superior sagittal sinus, which provides protective stabilization in most forms of trauma. Nevertheless, a direct injury to the frontal table can also result in a corresponding venous injury.

#### Middle cranial fossa

The sphenoid and temporal bones form the MCF. Its boundaries are defined by tuberculum sellae and the lesser wing of sphenoid anteriorly, the greater wing of sphenoid and squamous temporal bone laterally, and dorsum sellae and petrous ridge posteriorly. It comprises a central and lateral compartment, divided by an arbitrary line known as the petrous-clinoid line, connecting the tip of the anterior clinoid process to the petrous apex [14]. The central compartment contains the sella turcica and the lateral compartment houses the temporal lobe. The MCF contains multiple canals, foramina, and grooves housing many important neurovascular structures. In the context of this review, we will primarily focus on the relevant vascular structures in this region, and specifically the internal carotid artery (ICA) and the cavernous sinus.

The carotid canal is located within the petrous temporal bone of the MCF, which houses the petrous segment of the ICA (C2). The petrous segment of ICA is encased within the periosteum of the carotid canal. This segment has three parts: a vertical portion, a bend, and a horizontal portion [15, 16]. Along its course, the petrous ICA travels anteromedial to the tympanic cavity at the bend and medial to the eustachian tube at the horizontal portion. The petrous segment ends along the posterior foramen lacerum inferomedial to the edge of Meckel’s cave at the terminus of the carotid canal [16]. The lacerum segment (C3) begins at the end of the carotid canal, courses above without traversing the foramen lacerum, and ascends along the vertical canal of foramen lacerum. The lacerum segment ends at the superior margin of the petrolingual ligament, a continuation of the periosteum of the carotid canal. It enters the posterior cavernous sinus, where it becomes the cavernous segment.

The cavernous segment (C4) comprises a vertical portion, a posterior bend, a horizontal portion, and an anterior bend. The C4 segment typically gives rise to the meningohypophyseal trunk posteriorly, the inferolateral trunk laterally to the horizontal portion of the C4 segment. The C4 segment ends at the proximal dural ring formed by the junction of the medial and inferior periosteum of the anterior clinoid process [16].

The clinoid segment (C5) is a short segment coursing between the proximal and distal dural ring craniocaudally. It traverses between the anterior clinoid process and the carotid sulcus of the basisphenoid. Upon exiting the dural ring and entering the subarachnoid space, it becomes the ophthalmic segment (C6). Within the intradural C6 segment, it commonly gives rise to the ophthalmic artery and the superior hypophyseal artery [16, 17]. The communicating segment (C7) begins just proximal to the origin of the posterior communicating artery (PComA) and ends at the ICA bifurcation of the anterior cerebral artery and middle cerebral artery. Along the course of the C7 segment, it gives rise to the PComA and the anterior choroidal artery [16].

The cavernous sinuses are a pair of interconnected networks of venous channels within the central MCF that extend from the superior orbital fissure to the petrous apex. The cavernous sinuses are bounded by meningeal and periosteal dura layers; the medial wall, being the weakest, consists of a single dural layer, while the other walls comprise both layers [18]. The cavernous sinus houses the ICA and its associated sympathetic plexuses, as well as cranial nerves II, III, IV, and V1 [19]. The cavernous sinus drains the superior and inferior ophthalmic veins, pterygoid plexus, Sylvian vein, and sphenoid parietal sinus into the superior and inferior petrosal sinuses.

#### Posterior cranial fossa

The PCF houses the brain stem and cerebellum and is formed by the occipital bone and temporal bones. The anatomical boundaries are defined by the clivus, which forms the anteromedial boundary; the posterior edge of the petrous temporal bone, which forms the anterolateral boundary; the squamous occipital bone, which constitutes the posterior boundary; and the mastoid temporal bone, squamous, condylar, and basilar parts of the occipital bone, forming the floor of the PCF.

The vertebral arteries enter the dural ring through the foramen magnum and commonly give rise to the posterior inferior cerebellar artery (PICA). Recognition of the variant anatomies of PICA is important for accurate identification of commonly encountered injuries in clinical practice, and include extradural PICA, PICA as a terminal branch of the vertebral artery, PICA with the basilar artery origin, and multiple PICA branches. Another common variant is the AICA-PICA variant, in which AICA supplies the distal PICA territory in the absence of PICA and vice versa [20]. At the foramen magnum, the distal vertebral arteries typically supply the anterior spinal arteries, posterior spinal arteries, and posterior meningeal artery, though tremendous variability of anatomy exists [21]. Injuries involving the intradural vertebral artery at the skull base are associated with occipital condyle fracture (OCF), and in particular, occipital condyle avulsion fracture (Type III OCF) [6].

The sigmoid sinus courses along the sigmoid groove in the occipital bone, which drains into the internal jugular vein in the jugular foramen at the proximal end of the sigmoid groove. The jugular foramen sits in the posterior aspect of the petro-occipital suture and is separated anteriorly from the carotid canal by the caroticojugular spine. The jugular foramen sits lateral to the hypoglossal canal, separated by the jugular tubercle [22]. The jugular foramen is divided into two compartments by the jugular spine: the anteromedial compartment known as the par nervosa, houses the inferior petrosal sinus, and glossopharyngeal nerve (CN IX), and the posterolateral compartment, known as the par vascularis, contains the jugular bulb, vagus nerve (CN X) and spinal accessory nerve (CN XI) [22]. The transverse-sigmoid sinuses and internal jugular vein are at risk of developing dural venous sinus thrombosis (DVST) as a result of the adjacent skull base fracture.

### Screening criteria

Imaging is an essential part of trauma assessment, especially when evaluating skull base injuries. These injuries often result from high-energy mechanisms, and patients may be obtunded, which limits effective clinical assessment. In addition, there can be a delay between the injury and the onset of symptoms, and up to 80% of BCVI cases begin without initial symptom [23, 24]. Symptoms related to skull base injuries, including unexplained focal neurological deficits and arterial epistaxis, have high associations with blunt cerebrovascular injury (BCVI) (38–100%) [24–26] and warrant further imaging assessment to pursue the diagnosis [25]. Screening asymptomatic patients at risk for BCVI is more controversial. However, the majority of data support the recommendation that patients at risk for BCVI can be identified before the onset of symptoms with the appropriate screening [13, 26–29].

Several sets of criteria have been put forth to screen for BCVI in at-risk patients. These include Modified Denver Criteria, Memphis Criteria, and Boston Criteria (Tables 3, 4 and 5) [30–32]. Of these criteria, the modified Denver Criteria [33] have undergone the most extensive study and are widely used. The criteria have been endorsed by both the EAST and the Western Trauma Association [13, 34, 35]. Recent studies have suggested broadening the screening criteria to encompass additional clinical and radiological risk factors with suggested expansion including thoracic injuries, scalp degloving, thoracic vascular injuries, blunt cardiac rupture, and upper rib fracture (ribs 1–6) [36–38].

**Table 3:** Modified Denver Criteria for BCVI

Modified Denver Criteria for BCVI	
Clinical signs and symptoms	• Arterial hemorrhage • Cervical bruit • Expanding cervical hematoma • Focal neurological deficit • Neurological examination inconcordant with CT findings • Ischemic stroke on secondary CT
Risk factors for BCVI	• High-energy mechanism • Lefort II and III maxillofacial fractures • Cervical spine fracture patterns: subluxation, fracture extending to the foramen transversarium, fractures of C1-3 • Basilar skull fracture with carotid canal involvement • Diffuse axonal injury with Glasgow Coma Scale score ≤ 6 • Near hanging with anoxic brain injuries • Seat belt abrasion with significant swelling, pain, or altered mental status

**Table 4:** Memphis Criteria

Memphis criteria for BCVI	
• All cervical spine fractures • Neurologic examination inconcordant with CT findings • Horner syndrome • LeFort II and III maxillofacial fractures • Skull base fractures involving the foramen lacerum • Neck soft-tissue injury (e.g., Seat belt injury or hanging)	

**Table 5:** Boston Criteria

First Tier – CTA screening on presentation	• Skull base fractures: petrous and basilar fractures • Any cervical spine fractures • Cervical spine injury (Cord or ligaments) • Sof-tissue injury to anterior neck with swelling, ecchymosis, hematoma, and/or bruit • Significant neurologic deficit: lateralizing neurologic deficit, TIA, Horner syndrome • Evidence of stroke on CT
Second Tier – CTA screening within 24–48 h of presentation	• Diffuse axonal injury • Complex facial fractures with midface instability • Combined significant head and chest trauma • Near-hanging • Seat belt abrasion on the neck • Other unexplained neurologic deficits: vertigo, tinnitus, or Glasgow Coma Scale score ≤ 6

### Imaging modalities

The initial trauma imaging assessment starts with non-contrast CT of the head and cervical spine. Multidetector CT, with its rapid acquisition and wide availability, is the primary imaging modality in the initial evaluation of the skull base trauma. Reformatted images in orthogonal planes in bone and soft tissue kernels after initial thin section volumetric acquisition in the axial plane offer anatomic evaluation and assessment of soft tissue and osseous injuries with high sensitivity and specificity.

The imaging evaluation of traumatic cerebrovascular injuries encompasses various modalities, including CTA, MRI/MRA, or DSA. CTA head and neck, with its fast scan time and wide availability, is the primary imaging choice for initial screening and diagnosis of BCVI. CTA provides high spatial and contrast resolution images of arterial lumen and wall. The utilization of ≥ 16 slice multidetector CTA is recommended for identifying BCVI according to most guidelines [36]. CTA trauma assessment requires scanning of the entire length of the carotid and vertebral arteries from the aortic arch to the circle of Willis. Automatic bolus tracking techniques are commonly employed to optimize the timing of contrast injection. At our institution, CTAs are performed with 64-slice CT scanners, acquiring source images with a 0.625 mm slice thickness. Post-processing images for comprehensive vessel assessment include multiplanar thin slab maximum intensity projection (MIP) reconstructions, curved planar reformats, and 3D rotational MIP reconstructions.

CTAs are highly accurate in identifying BCVI with sensitivities of 64–98% and specificities of 92–100% compared to DSA [39–42]. The diagnostic accuracy of CTA may be limited by artifacts such as volume averaging, motion, beam-hardening, and less-than-optimal timing of the contrast bolus. However, CTA can effectively identify most clinically significant vascular injuries. False-negative results are more typical of low-grade vessel injuries with lower reported incidence of subsequent ischemic stroke [43].

Although MRI/MRA has generally been excluded as a screening tool for detecting BCVI due to the long scanning times, limited availability, and high relative cost [44], it can provide complementary diagnostic values in patients with suspected BCVI. Standard MRI provides more sensitive means for identifying acute cerebral infarction in the setting of suspected cerebrovascular injury and additional diagnostic information of related intracranial trauma such as diffuse axonal injury. The time-of-flight (TOF) technique can assess the patency of cerebrovascular structures without the need for intravenous contrast. Axial T1 sequence with fat saturation allows for accurate assessment of subacute intramural hematoma, which is characterized by crescent-shaped intrinsic T1 hyperintensity. MRA generally has lower diagnostic accuracy for identifying BCVI when compared to CTA and DSA. The reported sensitivity of MRA for detecting BCVI is between 50 and 75% and a specificity of 67% compared to DSA [41, 43, 45]. MRA is generally not recommended as the sole modality for screening BCVI [13].

Vessel Wall Imaging (VMI) can serve as a supplementary imaging technique, potentially enhancing diagnostic accuracy in high-risk patients by providing additional diagnostic insight in cases where differentiation between intracranial arterial dissection, atherosclerotic plaque, vasculitis, and other causes of arterial luminal narrowing is equivocal or challenging. VWI utilizes CSF and luminal blood signal suppression techniques to better delineate intracranial arterial vessel walls. This technique requires high spatial resolution and high signal-to-noise ratio and is therefore best suited for 3T scanners over scanners of lower field strength [46]. Intracranial arterial dissection on VWI is characterized by a curvilinear T2 hyperintense intimal flap separating the true and false lumens or an intramural hematoma with eccentric arterial wall thickening commonly containing intrinsic T1 hyperintensity typical of early subacute blood product [46].

DSA has superior spatial and temporal resolution for vascular imaging and is considered the gold standard for diagnosis of BCVI, though it has certain limitations. It is an invasive procedure that may not be feasible to perform in patients with additional critical traumatic injuries. Interventional radiology procedures require more time than noninvasive imaging and may result in a delay in diagnosis. Furthermore, it provides no information regarding vessel wall hematomas. In most cases, DSA is reserved for confirmatory and adjunct imaging modality in patients with equivocal CTA findings, and in cases where endovascular repair is anticipated.

Duplex ultrasound has low sensitivity for detecting BCVI (38.5-86%) and a limited acoustic window for assessing vessel injuries at the skull base [34, 40]. Vascular ultrasound is not a recommended imaging modality for evaluating BCVI by EAST guidelines [13].

### The spectrum of vascular complications from skull base trauma

#### Anterior cranial fossa dural arterial venous fistulas (ACF-dAVFs)

Fractures through the cribriform plate with extension to the anterior or posterior ethmoidal foremen may result in vessel injuries to the branches of ophthalmic arteries, specifically the anterior and posterior ethmoidal arteries. A known complication is the formation of anterior cranial fossa dural arterial-venous fistulae (ACF-dAVFs) [47, 48]. In ACF-dAVF, fistulization frequently occurs at the cribriform plate, where the afferent arterial blood supply arises from the distal ophthalmic artery and both anterior and posterior ethmoidal arteries. The venous return is typically through the frontal cortical veins, which drain into the superior frontal sinus or posteriorly into the cavernous sinus or basal vein of Rosenthal [49, 50].

**Fig. 1:**
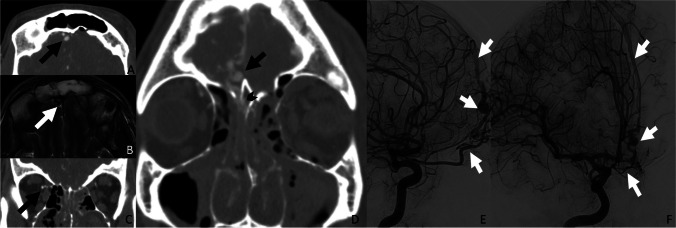
ACF dAVF. A 45-year-old male presented with severe craniofacial trauma involving the frontobasal distribution with skull base fractures extending along the ethmoid plates and left sphenoid sinus wall. The initial CTA demonstrated enlarged cortical veins along the right anterior cranial fossa (**A**) and prominent right ophthalmic and ethmoidal arteries (**C**). The MRI showed prominent flow voids of the enlarged cortical veins (**B**). On the CTA, the fistula point (black arrowhead) is centered at the fractured cribriform plate (**D**). Lateral and oblique internal carotid artery injections on the initial DSA demonstrated a fistulous connection between the ethmoidal branches of the ophthalmic artery with early venous drainage into the superior sagittal sinus (**E** and **F**)

ACF-dAVFs often demonstrate enlarged cortical veins along the cribriform plates (Fig. 1). In some cases, varices of adjacent cortical veins may also be present. MRI often demonstrates flow voids of enlarged cortical veins in the ACF as evidence of dAVF. DSA is the gold standard for diagnosing dAVF and typically identifies the presence of arterial-venous shunting from ethmoidal branches of the ophthalmic artery to enlarged cortical veins.

ACF-dAVF is known to carry a significant risk of intracranial hemorrhage due to venous drainage through fragile cortical pial veins. High-grade lesions are typically treated with trans-arterial, transvenous, or combined endovascular techniques. Surgical resection is generally reserved for difficult-to-access aneurysm locations. For low-grade lesions and those not amenable to endovascular or surgical treatment, conservative management with surveillance imaging is often employed.

### Post-traumatic ICA dissection

Carotid artery injury has been reported in approximately 1% of patients with blunt head trauma [26, 28]. The blunt force in a skull base trauma induces a differential change in momentum along the osseous structures and more elastic vascular structures. The abrupt immobility of the ICA as it enters the skull base at the carotid canal is a recognized area of vulnerability [51]. Once shearing is initiated, the energy leads to the separation of the vascular layers, resulting in dissection by the penetration of blood into the arterial wall through a primary tear [52]. The dissection typically extends downstream in the direction of the blood flow, with blood accumulating in the subintimal layer (between the intima and media) or the subadventitial layer (between the media and adventitia) [53]. The intramural hematoma in ICA dissection typically results in a narrow eccentric lumen with an increased external caliber of the artery, which may cause vessel stenosis or occlusion.

Clinical manifestations of ICA dissection vary depending on the degree of luminal narrowing and the presence of thromboembolism. High-grade stenosis, occlusion, or thromboembolic events arising from vessel injury are more likely to lead to focal neurological deficits from ischemic infarction, whereas low-grade narrowing can manifest with localized or transient symptoms. Local symptoms include head, facial, or neck pain, Horner syndrome, pulsatile tinnitus, and cranial nerve palsy [54, 55]. It is worth noting that up to 80% of patients with BCVI have latent clinical manifestations from the time of the injury, and it is during this period that imaging provides the highest value for diagnosing and guiding clinical management [23].

**Fig. 2:**
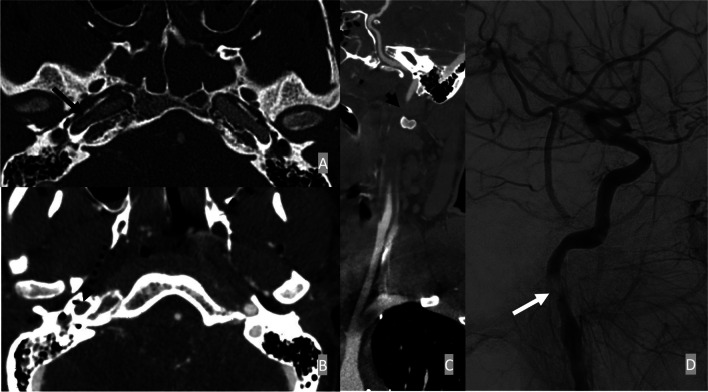
Petrous ICA dissection. A 19-year-old male presents after MVC with left parietal open depressed skull fracture, nondisplaced right temporal bone fracture, and diffuse SAH. The initial CTA demonstrated vessel irregularity of the proximal right petrous ICA (**A**). There was an absence of contrast opacification from the proximal cervical ICA to the proximal petrous ICA (**B** and **C**). Right lateral ICA injection of DSA (**D**) demonstrated Biffl Grade II flow-limiting dissection of the right petrous ICA

Common imaging features in CTA for arterial dissections include intraluminal narrowing with crescentic mural thickening, the presence of an intimal flap, and vessel irregularities with luminal narrowing (Fig. 2). In MRI, the intramural hematoma often exhibits changes in signal intensity as a result of the paramagnetic effect caused by the breakdown of hemoglobin in evolving hemorrhage [52]. The subacute phase, which contains methemoglobin, typically demonstrates high intrinsic T1 signal intensity, best shown on the fat-saturated T1-weighted sequence. The subacute intramural hematoma is, therefore, characterized by a peripheral, often eccentric rim of T1-signal hyperintensity encircling the flow void of the vessel lumen. DSA is considered the gold standard for diagnosing dissection due to its superior special and temporal resolution. Intimal flap, “string sign”, double lumen, and “flame sign” are common findings in DSA.

Patients with low-grade dissections are typically treated conservatively with close observation and anticoagulation if not otherwise contraindicated. Endovascular treatment for dissection is reserved for patients with flow-limiting/occlusive vascular lesions, associated expanding or symptomatic pseudoaneurysm (discussed below), and contraindications to anticoagulation such as intracranial or systemic hemorrhage. The treatment goals are to minimize the progression of vessel injury, decrease the incidence of ischemic events, and improve overall neurologic and survival outcomes [51].

#### Post-traumatic aneurysms

**Fig. 3:**
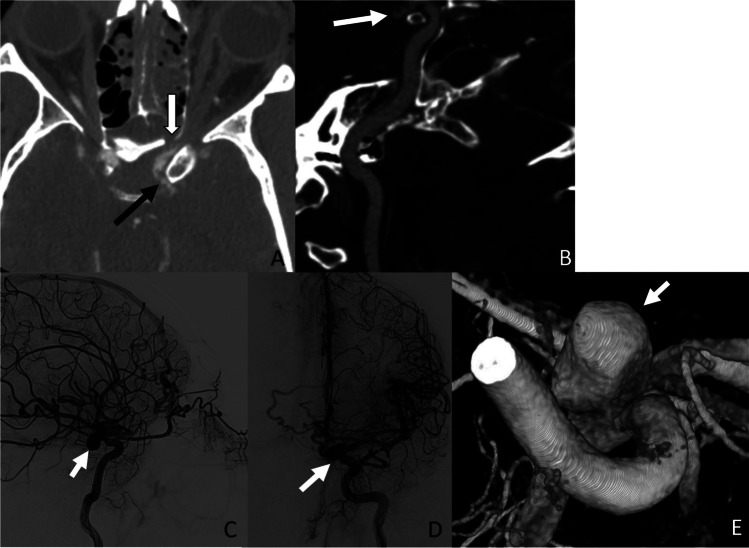
Post-traumatic aneurysm. A 45-year-old male presented with severe craniofacial trauma involving the frontobasal distribution after an ATV rollover. The initial CTA neck demonstrated vessel irregularity and non-flow limiting stenosis along the left supraclinoid ICA adjacent to the sphenoid sinus wall fracture (**A** and **B**). The left ICA injections in the lateral, frontal projections, and 3D reformats on the follow-up DSA demonstrated the development of a medially projecting ICA sidewall pseudoaneurysm (**C**, **D**, and **E**)

Traumatic intracranial aneurysms (Fig. 3) are rare, accounting for < 1% of all aneurysms [56, 57]. Post-traumatic aneurysms can be classified as either true aneurysms or false aneurysms. True aneurysms typically arise from a partial disruption of the vessel wall (internal elastic lamina, intima, and media) with intact adventitia, whereas false aneurysms or pseudoaneurysms result from the complete disruption of the vessel wall with the development of contained hematoma restricted by the adjacent perivascular connective tissue [58]. The mortality rate of traumatic aneurysm may be as high as 50% due to intracranial hemorrhage and delayed rupture [59]. Early detection followed by prompt endovascular or surgical intervention is critical for effective treatment [60]. Clinical presentations of post-traumatic aneurysm vary depending on the location, size, and bleeding status, and may include epistaxis, headaches, seizures, and neurological deficits [58].

Traumatic pseudoaneurysms, by definition, are contained previously ruptured aneurysms, and carry a higher risk for rupture and re-bleeding due to weakened or incomplete vessel walls. The reported risk of hemorrhage in posttraumatic pseudoaneurysm is 19%, with the highest incidence of rupture occurring at 2–3 weeks following the initial injury [61, 62].

Post-traumatic pseudoaneurysm potentially serves as a source for thromboembolism. This is mainly attributed to the abnormal flow dynamics within the aneurysmal sac, coupled with exposed subendothelial thrombogenic materials that promote blood coagulation, leading to downstream thrombus embolization [63]. When large enough, pseudoaneurysm may exert a mass effect on the adjacent vessel wall, resulting in luminal stenosis [64].

When identified, pseudoaneurysms are typically treated with endovascular techniques, including coil embolization, stent-assisted coil embolization, flow diversion, or parent artery occlusion. In cases where endovascular treatment proves ineffective or if the pseudoaneurysm is in a location that cannot be accessed easily, surgical intervention is employed through the use of clipping, wrapping, bypass, or ligation of the parent artery.

#### Carotid-cavernous fistula

A carotid-cavernous fistula (CCF) is an acquired abnormal arterial-venous shunt between the cavernous ICA or ICA branch and the adjacent cavernous sinus. CCFs can be classified angiographically into direct (Barrow type A) and indirect or dural (Barrow type B, C, and D) types Direct CCFs (Barrow type A) typically exhibit high-flow hemodynamics. They can be secondary to skull base trauma, iatrogenic carotid injury, or spontaneous rupture of a cavernous ICA aneurysm [65, 66]. Indirect (dural) CCFs are abnormal arterial-venous shunting connections between cavernous sinus and branches of ICA (Barrow type B), branches of ECA (Barrow type C), or both (Barrow type C) [67]. Indirect CCFs are believed to be the result of cavernous venous thrombosis and typically exhibit low-flow hemodynamics [68].

Patients with direct CCFs often present with rapid development and progression of symptoms due to high-flow arteriovenous hemodynamics. Clinical symptoms may include proptosis, chemosis, red eye from arterialization of the intraorbital veins, strabismus from orbital congestion and/or cranial nerve palsy, orbital bruits from high-flow fistula, and increased intraocular pressure [65]. Intraparenchymal or subarachnoid hemorrhage may occur due to cortical venous congestion, affecting approximately 5% of patients [69]. Patients with indirect fistula generally have more indolent presentations because of relatively low-flow hemodynamics [66].

The diagnosis of CCF can be confirmed through neuroimaging if clinically evident or otherwise suspected. CTA and MRA are accurate in diagnosing CCF with a sensitivity of 87% and 80%, respectively, although DSA has a superior sensitivity of 94.4% [70].

**Fig. 4:**
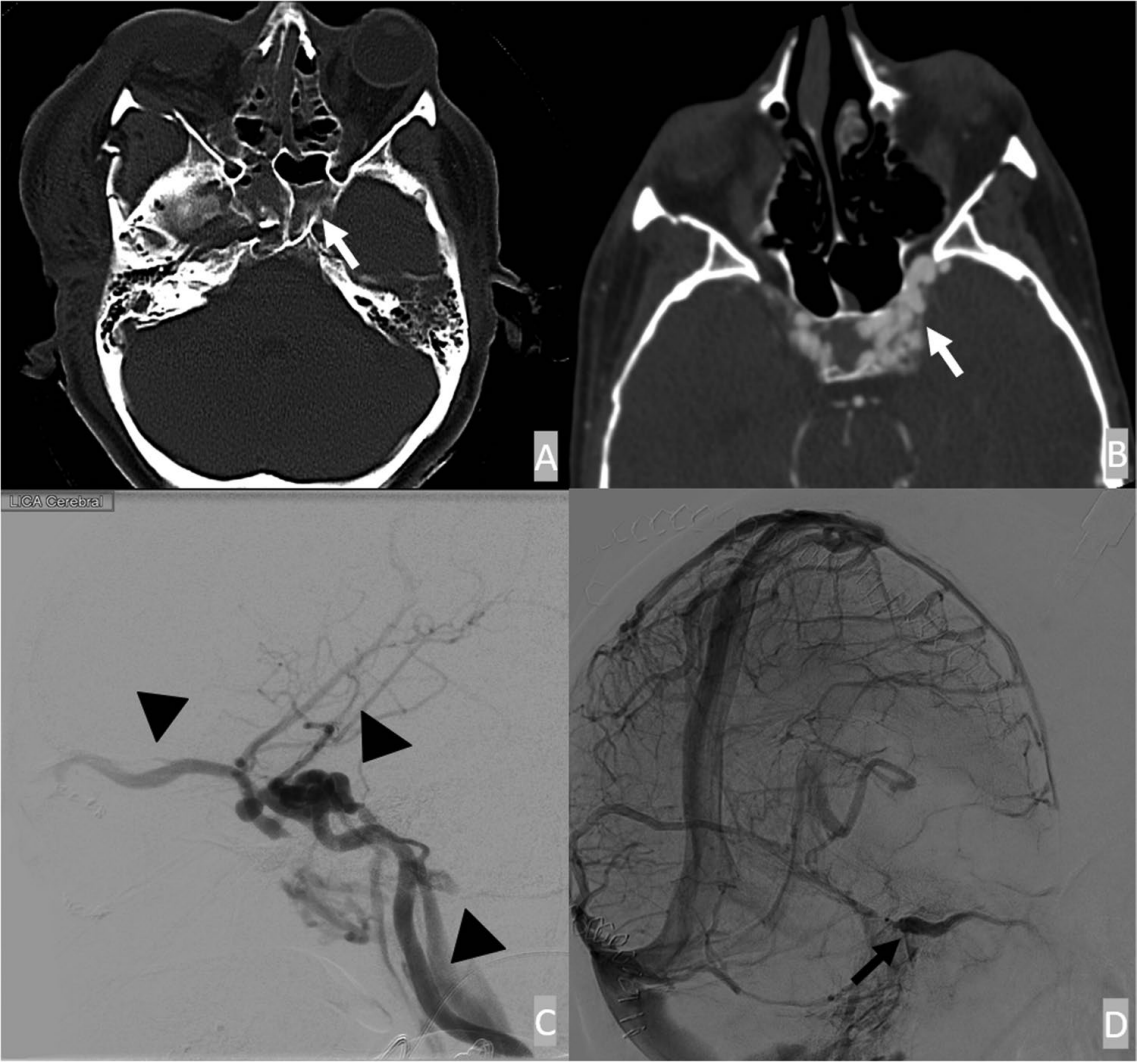
Carotid-cavernous fistula (CCF). A 47-year-old female presented after a motor vehicle collision with extensive craniofacial fractures including a fracture involving the left sphenoid sinus wall (**A**). Four months later, the patient presented with pulsatile tinnitus and exophthalmos. Axial CT head angiogram (**B**) demonstrated bulging and asymmetric enhancement of the left cavernous sinus, compatible with a carotid-cavernous fistula. The patient subsequently underwent DSA. The lateral projection of arterial phase (**C**) showed shunting from the left internal carotid artery to the cavernous sinus and the enlarged draining veins. There was retrograde flow from the cavernous sinus into the ophthalmic vein (arrowheads). The venous phase of the internal carotid artery in oblique projection (**D**) demonstrated prolonged filling of the superior ophthalmic vein (black arrow)

CTA and MRA typically demonstrate an enlarged superior ophthalmic vein, bulging of the cavernous sinus, and sometimes a fistula tract from the internal carotid artery. Orbital findings typically include proptosis, retrobulbar edema, and extraocular muscle enlargement because of orbital congestion. DSA typically demonstrates rapid shunting from ICA to cavernous sinus and retrograde flow from cavernous sinus draining into the ophthalmic veins (Fig. 4).

The treatment of CCF depends on the severity of clinical symptoms, angiographic characteristics, and risk for intracranial hemorrhage. Conservative medical management is an acceptable approach for treating patients with non-emergent ocular symptoms as dural fistulas may undergo spontaneous resolution within days to months [71]. The endovascular approach has emerged as the primary treatment choice during clinical emergencies and in cases where conservative therapy has proven unsuccessful. The objective of treating direct carotid-cavernous fistulas (CCFs) is to close the tear between the ICA and the cavernous sinus while maintaining the patency of the ICA. This can be achieved through trans-arterial obliteration via a detachable balloon, trans-arterial or transvenous embolization to obliterate the ipsilateral cavernous sinus, or deployment of a covered stent across the fistula. Indirect CCFs are most commonly treated by transvenous embolization or by trans-arterial embolization of the arterial branches supplying the fistula [71].

#### Vertebral artery injury from occipital condyle fractures

Blunt vertebral artery injury (BVAI) occurs in 0.5-2% of all blunt trauma and carries an overall mortality rate of 4–8% [72–74]. High-risk factors include C1-2 fractures, foramen transversarium involvement, subluxation, and type III OCF. BVAI may lead to stenosis, occlusion, pseudoaneurysm, and AVF. Although often asymptomatic due to adequate collateralization, patients who develop vertebral artery ischemia may suffer devastating neurological deficits. In cases where the basilar artery is involved, the mortality rate has been reported to be as high as 80% [73]. Symptomatic BVAI is due to posterior circulation ischemia of the brainstem and cerebellum, which may result in headache, neck pain, sensory and gait disturbance, dizziness, nausea, vomiting, speech and visual deficits [74].

Injuries to the skull base, particularly type III OCF, can exert substantial translational force, placing strain on the vertebral arteries. According to the Anderson and Montesano classification, OCFs are categorized into Types I – III. Type III OCF is characterized by an avulsion fracture of the occipital condyle within the region of the alar ligament. This fracture type is commonly a result of significant translational force, which can lead to direct injury of the intradural segment of the vertebral artery. Type III OCF has been identified as an independent risk factor for BVAI [6].

**Fig. 5:**
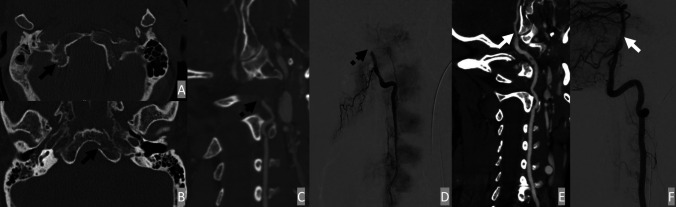
Vertebral arteries dissection from occipital condyle fractures. A 53-year-old male presented with bilateral occipital condyle fractures after MVC (**A** and **B**). The right vertebral artery curve reformat from the initial CTA neck (**C**) demonstrates abrupt tapering with the absence of contrast opacification of the V3 and V4 segments, compatible with flow-limiting dissection. The right vertebral artery injection of DSA (**D**) demonstrated occlusion of the V3 segment. The left vertebral artery curve reformat from the CTA neck (**E**) showed vessel irregularity of the left V4 segment. Left vertebral artery injection of DSA (**F**) demonstrated a short segment of non-flow limiting dissection

Blunt cerebrovascular injury usually presents 10–72 h after the initial trauma. Screening for BVAI in this time frame provides optimal value, guiding treatment planning with consideration for anticoagulation to prevent delayed presentation leading to ischemic events arising from the posterior circulation. Moreover, upon identifying BVAI lesions, close follow-up can be initiated to ensure the resolution of the lesion and to detect any potential late complications, such as pseudoaneurysm formation or arteriovenous fistula. As previously discussed, CTA is the primary imaging modality for screening TVAI. Common imaging findings of BVAI often include intraluminal narrowing with crescent mural thickening, irregular “rat’s tail” stenosis, double lumen, and segmental occlusion (Fig. 5).

The options for treatment for BVAI include supportive management (most common), anticoagulation, and endovascular management [6]. Intraluminal stents are employed to treat persistent pseudoaneurysms, flow-limiting stenosis, or occlusion. In cases of transected vessels, arterio-venous fistulae, or pseudoaneurysms not amenable for stenting, other endovascular techniques, including embolization or balloon occlusion, may be considered. Due to the locations of these injuries, open repair is not feasible for the majority of patients [75]. Although the optimal timing remains undetermined, follow-up CTA may be performed to characterize evolution and possible resolution of the vascular lesions. Typically, BVAI radiographically evolves or resolves within 7–10 days, with some authors proposing a 7–14 days follow-up CTA to confirm resolution [6, 75].

#### Dural venous sinus thrombosis from skull base fracture

Dural venous sinus thrombosis (DVST) is an increasingly recognized condition in patients presenting with blunt traumatic brain injury and skull fractures [76]. When a skull fracture extends to a dural venous sinus or jugular bulb, it can lead to dural venous sinus thrombosis via direct mechanical disruption of the endothelium of the sinus wall [76]. Clinical presentations of DVST in the context of trauma can vary, and its clinical course may be insidious until subsequent complications arise, prompting medical attention. A meta-analysis conducted by Bokhari et al. revealed that 26% of patients with blunt head trauma and skull fractures extending to a dural venous sinus or jugular bulb developed DVST [77]. Venous sinus occlusion was reported in 55% of DVST in the setting of trauma [78]. Venous infarctions were reported in 7–38% of Traumatic DVST cases [76].

**Fig. 6:**
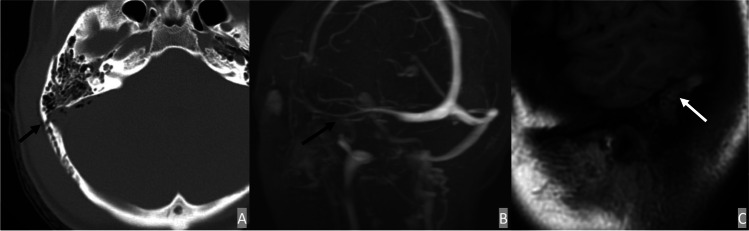
Venous sinus thrombosis from fracture of sigmoid groove. A 57-year-old male presented with a fracture along the right temporal bone with resulting venous sinus thrombosis. Axial CT head (**A**) demonstrates fracture along the tympanic portion of the right temporal bone with resultant pneumocephalus. 3D reconstructed image of MRV (**B**) demonstrates a focal area of filling defect in the right transverse-sigmoid sinus, compatible with venous sinus thrombosis. Sagittal T1 image (**C**) shows a focal thrombus with intrinsic T1 hyperintensity

There is variability in the literature regarding screening protocols and indications for traumatic dural venous sinus thrombosis (DVST). It is generally established that skull base fracture and epidural hematoma overlying the dural venous sinus are independent risk factors for CVST development [79, 80]. When clinically suspected or suggested by initial trauma imaging, further evaluation via CT venogram (CTV) or MR venogram (MRV) is warranted. An acute thrombus typically exhibits hyperattenuation on a non-contrast CT with associated filling defect on CTV. In the acute phase on MRI/MRV, DVST typically exhibits T1 iso-hypointense and T2 hypointense and becomes T1 hyperintense in the subacute phase (Fig. 6).

The existing data on therapeutic management of dural venous injury and thrombosis is limited. In contrast to spontaneous CVST, therapeutic management of traumatic DVST requires a careful balance between the risk of thrombus progression and the risk of traumatic hemorrhage, with decisions made on an individualized, case-to-case basis informed by the severity of symptoms and in consideration of co-existing injuries basis [76]. The outcome of anticoagulation in this patient population at risk for hemorrhage also remains uncertain [77].

## Conclusion

Trauma to the skull base can lead to cerebrovascular complications with immediate and delayed clinical and radiological manifestations with potentially devastating outcomes. With a comprehensive understanding of the imaging anatomy and pathophysiology of skull base trauma, imaging techniques, clinical presentation, screening criteria, and treatment options, radiologists are better equipped to provide accurate and timely diagnoses and recommendations to guide clinical management.
